# Crystal structures of organoplatinum complexes containing alkyl­eugenoxyacetate and *p*-chloro­aniline

**DOI:** 10.1107/S2056989016008872

**Published:** 2016-06-10

**Authors:** Hai Le Thi Hong, Diep Dao Thi Bich, Ngan Nguyen Bich, Luc Van Meervelt

**Affiliations:** a Chemistry Department, Hanoi National University of Education, 136 - Xuan Thuy - Cau Giay, Hanoi, Vietnam; b KU Leuven - University of Leuven, Department of Chemistry, Celestijnenlaan 200F - bus 2404, B-3001 Heverlee, Belgium

**Keywords:** crystal structure, *trans*-di­chlorido­platinum(II) complexes, *p*-chloro­aniline, hydrogen bonding

## Abstract

In the title complexes, *trans*-di­chlorido­(4-chloro­aniline-κ*N*){3-meth­oxy-4-meth­oxy­carbonyl­meth­oxy-1-[(2,3-η)-prop-2-en-1-yl]benzene}­platinum(II) 0.1-hydrate, [PtCl_2_(C_6_H_6_ClN)(C_13_H_16_O_4_)]·0.1H_2_O, (I), and *trans*-di­chlorido­(4-chloro­aniline-κ*N*){4-eth­oxy­carbonyl­meth­oxy-3-meth­oxy-1-[(2,3-η)-prop-2-en-1-yl]benzene}­platinum(II), [PtCl_2_(C_6_H_6_ClN)(C_14_H_18_O_4_)], (II), the Pt^II^ metal atom displays a slightly distorted square-planar coordination geometry defined by the aniline N atom, two chloride anions (*trans*-positioned) and one ethyl­enic double bond. The least-squares planes through the two aromatic ring systems make an angle of 47.3 (3)° for (I) and 38.6 (2)° for (II). Both structures show disorder for the PtCl_2_ fragment, in the case of (I) even further extended towards the CH_2_—CH=CH_2_ ligand. An intra­molecular C—H⋯Cl hydrogen bond occurs in (I). In the crystal packing of (I), which is dominated by N—H⋯O and C—H⋯Cl inter­actions, a partially occupied water mol­ecule is observed on a twofold rotation axis with a refined site occupancy of 0.10 (1). A C—H⋯π inter­action is also present. In (II), inversion dimers form chains along the *b-*axis direction by N—H⋯O hydrogen bonds.

## Abstract 2

In the title *trans*-di­chlorido­platinum(II) complexes, the central Pt^II^ atom is further coordinated by the *p*-chloro­aniline N atom and ethyl­enic double bond of alkyl­eugenoxyacetate.

## Chemical context   

Complexes of platinum(II) such as cisplatin, carboplatin and oxaliplatin have been known to exhibit inhibitory activities on several human cancer cells and are widely used in pharmacy (Zhang *et al.*, 2006 ▸). However, many side effects and drug-resistant phenomena have been reported for the use of these complexes (Von Hoff *et al.*, 1979 ▸; Coates *et al.*, 1983 ▸; Griffin *et al.*, 1996 ▸). Therefore, it is necessary to design new complexes with high activities but low toxicity (Chabner & Roberts, 2005 ▸; Johnstone *et al.*, 2014 ▸). For this purpose, we have recently synthesized several Pt^II^ complexes containing natural aryl­olefines as ligand, *i.e.* derivatives of eugenol (4-allyl-2-methoxylphenol) such as methyl­eugenol and alkyl­eugenoxyacetate, with high toxicity towards human cancerous cells (IC_50_ values < 5 µg/mL; Da *et al.*, 2012 ▸; Da, Chi *et al.*, 2015 ▸; Da, Hai *et al.*, 2015 ▸). Inter­estingly, these complexes represent special arrangements in which the Pt^II^ atoms are coordinated by aryl­olefines through the C=C bond of the allyl group. Complexes of Pt^II^ containing methyl- or ethyl­eugenoxyl­acetate and *p*-chloro­aniline were synthesized and their crystal and mol­ecular structures characterized and reported here.
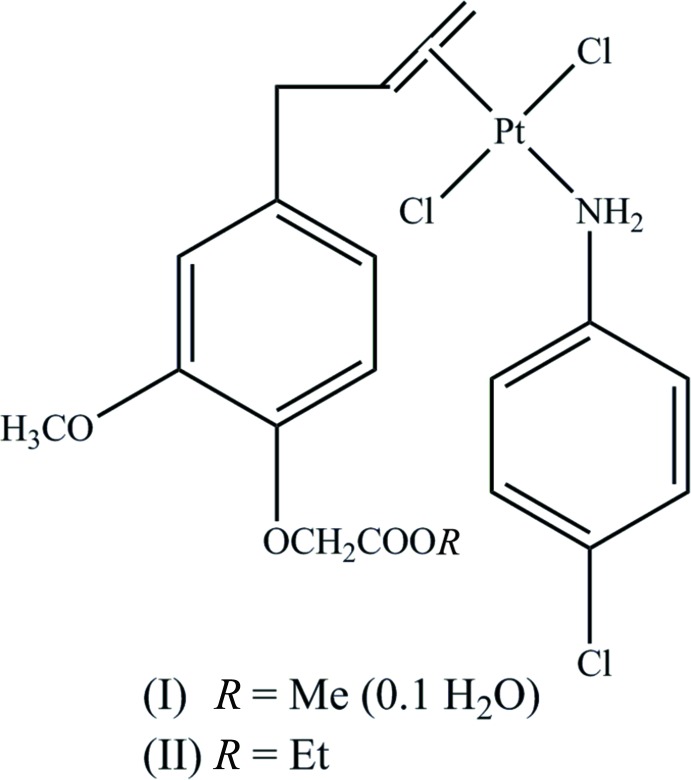


## Structural commentary   

**Figure 1:**
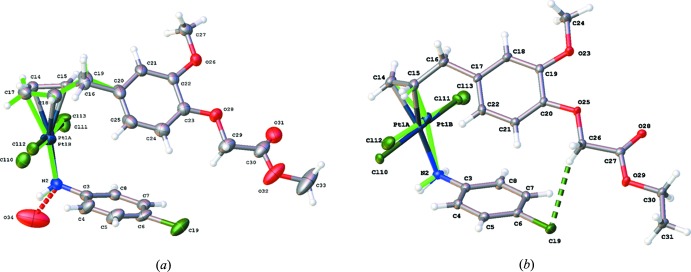
Views of the asymmetric units in (*a*) (I) and (*b*) (II), showing the atom-labelling schemes. Displacement ellipsoids are drawn at the 50% probability level. The intra­molecular C—H⋯Cl inter­action is shown as a green dotted line.

The complexes crystallize in different space groups, *C*2/*c* for the methyl­eugenoxyacetate derivative (I) and *P* 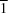 for the ethyl­eugenoxyacetate derivative (II). The central Pt^II^ metal atom displays a distorted square-planar coordination with the Pt^II^ atom coordinated by two Cl atoms, the NH_2_-group of *p*-chloro­aniline and the C=C double bond of the eugenol ligand. In both complexes, the Cl atoms are *trans* with respect to each other (Fig. 1 ▸). The eugenol ligand only inter­acts *via* the C=C double bond and not by a C atom of the phenyl ring. Both structures show some disorder of the Pt^II^ atom and its environment. In (I) the PtCl_2_CH_2_=CH—CH_2_ fragment is disordered over two positions [population parameters 0.679 (8) and 0.321 (8)], while in (II) only the PtCl_2_ fragment is disordered over two positions [population parameters 0.872 (6) and 0.128 (6)]. The angles between the best planes through the two aromatic rings are 47.3 (3) and 38.6 (2)° for (I) and (II), respectively. An intra­molecular C—H⋯Cl inter­action is observed for (II) with a H26*A*⋯Cl9 distance of 2.73 Å. In (I) the shortest intra­molecular H⋯Cl contact distance is 3.13 Å for H29*A*⋯Cl9.

## Supra­molecular features   

**Figure 2:**
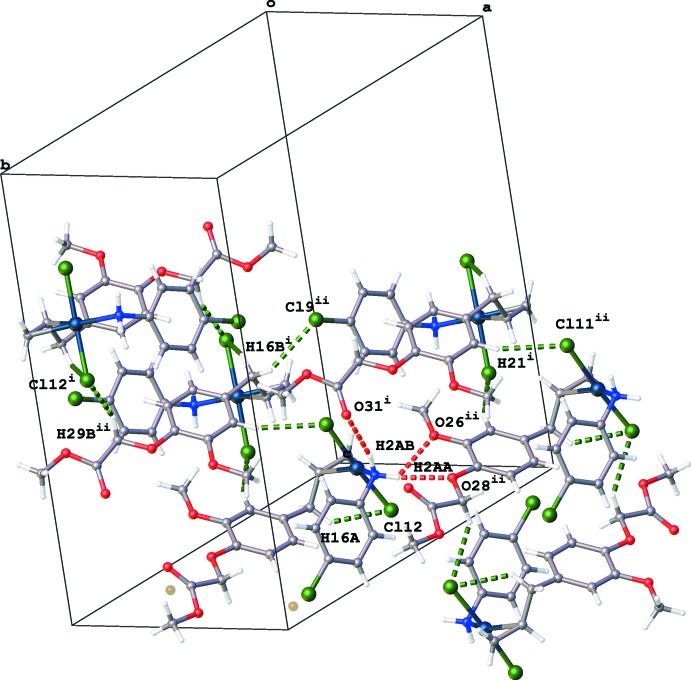
Partial packing diagram of (I), showing the N—H⋯O (red dotted lines) and C—H⋯Cl inter­actions (green dotted lines). [Symmetry codes: (i) *x* + 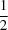, *y* − 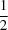, *z*; (ii) −*x* + 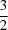, *y* − 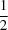, −*z* + 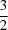; (iii) *x* − 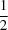, *y* − 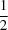, *z*; (iv) *x*, −*y* + 1, *z* − 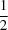.]

**Figure 3:**
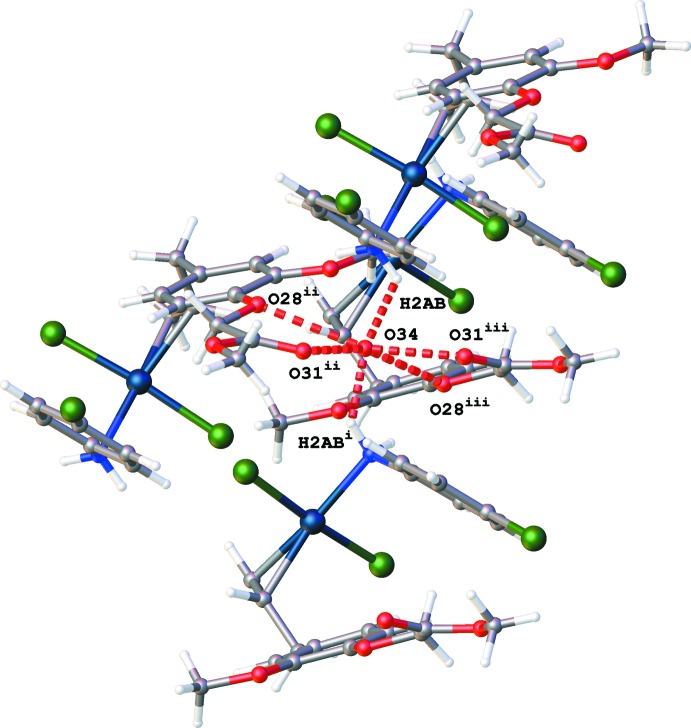
Partial packing diagram of (I), showing the inter­actions of water mol­ecule O34 with N2, O28 and O31 (red dotted lines). [Symmetry codes: (i) −*x* + 2, *y*, −*z* + 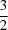; (ii) *x* + 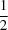, *y* − 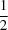, *z*; (iii) −*x* + 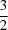, *y* − 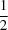, −*z* + 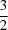.]

The crystal packing of (I) is built up by N—H⋯O and C—H⋯Cl inter­actions (Table 1 ▸, Fig. 2 ▸). A water mol­ecule O34 was identified at a special position [on a twofold rotation axis, occupancy factor 0.10 (1)] where it inter­acts with atoms N2, O28 and O31 linking four mol­ecules together (Fig. 3 ▸).

**Figure 4:**
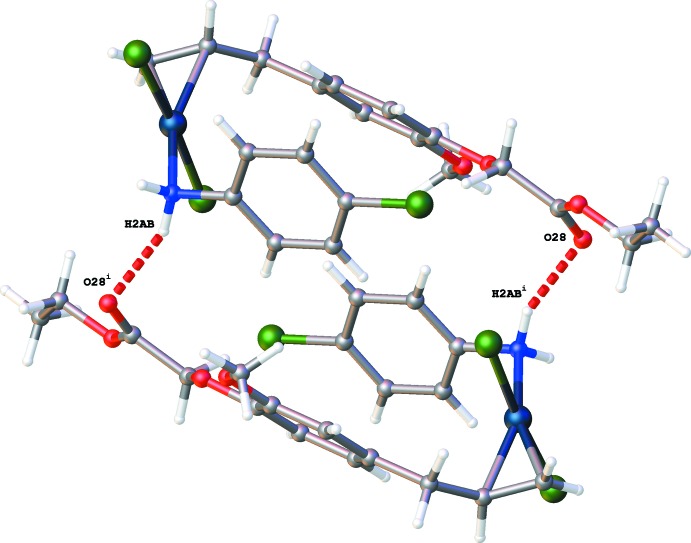
Formation of an inversion dimer by N—H⋯O hydrogen bonds drawn as red dashed lines in packing of (II). [Symmetry code: (i) −*x* + 1, −*y*, −*z* + 1.]

The crystal packing of (II) is dominated by hydrogen-bonding inter­actions (Table 2 ▸). Inversion dimers created by N2—H2*AB*⋯O28^i^ hydrogen bonds are further linked into chains in the *b-*axis direction by N2—H2*AA*⋯(O23^ii^,O25^ii^) hydrogen bonds [Figs. 4 ▸ and 5 ▸; symmetry codes: (i) − *x* + 1, −*y*, − *z* + 1; (ii) *x*, *y* + 1, *z*].

No π–π inter­actions are observed in the packing of either structure. For (I) a C—H⋯π inter­action is present [C27—H27*B*⋯*Cg*1^iii^, H27*B*⋯*Cg*1^iii^ = 2.72 Å; *Cg*1 is the centroid of the C20-C25 ring; symmetry code: (iii) −*x* + 1, *y*, −*z* + 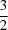].

## Database survey   

The Pt—N distances in (I) and (II) vary from 2.033 (6) to 2.273 (8) Å and deviate for the minor parts (Pt1*B*) due to the disorder from the average Pt—N distance of 2.09 (5) Å for Pt—NH_2_—phenyl fragments present in the Cambridge Structural Database (CSD, Version 5.37; Groom *et al.*, 2016 ▸). The Pt1*A*—Cl distances are between 2.288 (4) and 2.305 (2) Å and agree well with the average Pt—Cl distance of 2.32 (3) Å for *trans* complexes present in the CSD. One Pt1*B*—Cl distance [2.151 (2) Å] deviates significantly from this average.

A search in the CSD for Pt complexes with Pt coordinated by Cl, NH_2_ and C=C shows 11 hits. As fourth ligand we notice an additional Cl atom (eight hits, five *trans* and three *cis* coordinations) or C atom (two hits) or O atom (one hit). In the complex [PtCl(methyl­eugenol)(o-toluidine)] (CSD refcode GOYJEL; Da, Chi *et al.*, 2015 ▸), the central Pt atom coordinated by only one Cl atom, the NH_2_ group of *o*-toluidine, the C=C double bond of the eugenol ligand and also a C atom of the eugenol ligand. In (I) and (II) this last inter­action is not present and is replaced by an additional Cl atom.

## Synthesis and crystallization   

***Synthesis of K[Pt(Alkeug)Cl_3_]:***

The mononuclear complexes K[Pt(Alkeug)Cl_3_] (Alkeug are methyl­eugenoxyl­acetate or Meteug, and ethyl­eugenoxyl­acetate or Eteug) were synthesized following the protocol of Da and coworkers (Da *et al.*, 2012 ▸; Da, Chi *et al.*, 2015 ▸; Da, Hai *et al.*, 2015 ▸).

***Synthesis of trans-[PtCl_2_(Alkeug)(C_6_H_6_NCl)]:***

A solution of 127.0 mg (1.0 mmol) p-chloro­aniline in 10 mL acetone/ethanol (1:1 *v*/*v*) was added to a mixture of 1.0 mmol [K[Pt(Alkeug)Cl_3_] and 10 mL acetone/ethanol (1:1 *v/v*). After two h stirring, a white precipitate of KCl was separated out. The remaining solution was stirred for two h at room temperature to obtain a yellow precipitate, which was collected by filtration, washed with ethanol and diethyl ether and dried in vacuum. The obtained crystals are soluble in chloro­form and acetone, slightly soluble in ethanol and insoluble in water. The yield was 70–80%. Single crystals suitable for X-ray investigation were obtained by slow evaporation from a chloro­form/ethanol (1:2 *v/v*) solution at room temperature.

***Data for [PtCl_2_(Meteug)(C_6_H_6_NCl)] (I):***

IR (Impack-410 Nicolet spectrometer, KBr, cm^−1^): 3243, 3164 (ν_NH_); 3060, 2958, 2836 (ν_CH_); 1746 (ν_C=O_); 1592, 1517 (aromatic, ν_C=C_, ν_C=N_); 545 (ν_Pt-N_).

^1^H NMR (δ p.p.m; 500 MHz, CDCl_3_): 3.07 (*br*, 1H, –CH_2_­_a_); 3.40 (*dd*, ^2^ *J* = 15.0 Hz, ^3^ *J* = 8.0 Hz, –CH_2b_); 5.54 (*br*, 1H, all­yl); 4.54 (*br*, 1H, *cis*-alkene); 4.65 (*d*, *J* = 13.0 Hz, 1H, *trans*-alkene); 6.85 (*ov*, 1H, Ar); 6.83 (*ov*, 1H, Ar); 6.71 (*d*, *J* = 8.0 Hz, 1H, Ar); 3.82 (*ov*, 3H, –OCH_3_); 4.69 (*s*, 2H, –CH_2_–); 7.32 (*ov*, 4H, Ar), 6.10 (*br*, NH_2_).

***Data for [PtCl_2_(Eteug)(C_6_H_6_NCl)] (II):***

IR (KBr, cm^−1^): 3239, 3160 (ν_NH_); 3060, 2921, 2823 (ν_CH_); 1746 (ν_C=O_); 1595, 1516 (aromatic, ν_C=C_, ν_C=N_); 550 (ν_Pt-N_).

^1^H NMR (δ p.p.m; 500 MHz, CDCl_3_): 3.08 (*br*, 1H, –CH_2_­_a_); 3.39 (*dd*, ^2^ *J* = 15.5 Hz, ^3^ *J* = 7.5 Hz, 1H, –CH_2b_); 5.53 (*br*, 1H, all­yl); 4.63 (*d*, *J* = 9.5 Hz, 1H, *cis*-alkene); 4.52 (*br*, 1H, *trans*-alkene); 6.78 (*ov*, 1H, Ar); 6.82 (*d*, *J* = 8.0 Hz, 1H, Ar); 6.70 (*d*, *J* = 8.0 Hz, 1H, Ar); 3.82 (*s*, 3H, –OCH_3_); 4.28 (*q*, *J* = 7.0 Hz, 2H, –CH_2_); 1.30 (*t*, *J* = 7.0 Hz, 3H, –CH_3_); 4.67 (*s*, 2H, –CH_2_–); 7.30 (*ov*, 4H, Ar), 6.20 (*br*, NH_2_).

## Refinement   

Crystal data, data collection and structure refinement details are summarized in Table 3 ▸. Both structures show disorder which was modelled as good as possible, but still some larger peaks are present in the difference maps.

In (I) the PtCl_2_CH_2_=CH—CH_2_ fragment is disordered over two positions [population parameters 0.679 (8) and 0.321 (8)] and refined with constraints for the bond lengths present in this fragment. Refinement of the population parameter of oxygen atom O34 (at special position) converged to 0.10 (1). Water H atoms were not located.

In (II) only the PtCl_2_ fragment is disordered over two positions [population parameters 0.872 (6) and 0.128 (6)].

All H atoms were placed in idealized positions and refined in riding mode, with *U*_iso_(H) values assigned as 1.2*U*_eq_ of the parent atoms (1.5 times for methyl groups), with C—H distances of 0.95 (aromatic and =CH_2_), 0.98 (CH_3_), 0.99 (CH_2_) and 1.00 Å (CH), and N—H distances of 0.91 Å (NH_2_). Enhanced rigid bond restraints were used for the anisotropic temperature factors of the non-H atoms. In the final cycles of refinement, 7 and 15 outliers were omitted for (I) and (II), respectively.

## Supplementary Material

Crystal structure: contains datablock(s) I, II. DOI: 10.1107/S2056989016008872/rz5189sup1.cif

Structure factors: contains datablock(s) I. DOI: 10.1107/S2056989016008872/rz5189Isup2.hkl

Structure factors: contains datablock(s) II. DOI: 10.1107/S2056989016008872/rz5189IIsup3.hkl

CCDC references: 1483067, 1483066

Additional supporting information:  crystallographic information; 3D view; checkCIF report

## Figures and Tables

**Figure 5:**
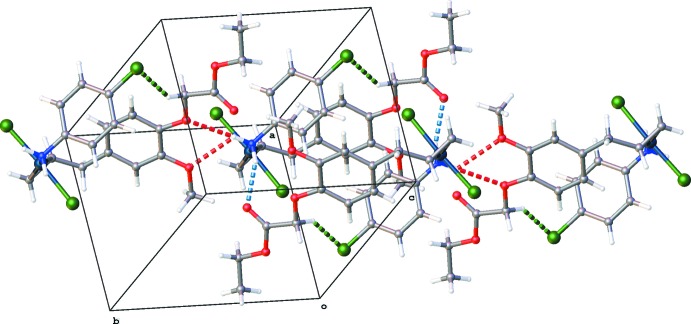
Chains of mol­ecules in the *b*-direction in the packing of (II) by N—H⋯O hydrogen bonds (red dashed lines). Other N—H⋯O and C—H⋯Cl inter­actions shown as blue and green dashed lines, respectively.

**Table 1:** Hydrogen-bond geometry (Å, °) for (I) *Cg*1 is the centroid of the C20–C25 ring.

*D*—H⋯*A*	*D*—H	H⋯*A*	*D*⋯*A*	*D*—H⋯*A*
N2—H2*AA*⋯O26^i^	0.91	2.45	3.027 (5)	122
N2—H2*AA*⋯O28^i^	0.91	2.35	3.212 (6)	158
N2—H2*AB*⋯O31^ii^	0.91	2.41	3.302 (3)	167
C16—H16*A*⋯Cl12	0.99	2.79	3.487 (10)	128
C16—H16*B*⋯Cl9^iii^	0.99	2.76	3.472 (8)	129
C21—H21⋯Cl11^iv^	0.95	2.82	3.726 (8)	159
C29—H29*B*⋯Cl12^v^	0.99	2.80	3.651 (7)	145
C27—H27*B*⋯*Cg*1^iv^	0.98	2.72	3.523 (6)	139

Symmetry codes: (i) 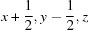; (ii) 
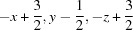
; (iii) 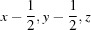; (iv) 
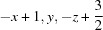
; (v) 
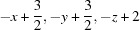
.

**Table 2:** Hydrogen-bond geometry (Å, °) for (II)

*D*—H⋯*A*	*D*—H	H⋯*A*	*D*⋯*A*	*D*—H⋯*A*
N2—H2*AA*⋯O23^i^	0.91	2.49	3.025 (5)	118
N2—H2*AA*⋯O25^i^	0.91	2.47	3.377 (5)	174
N2—H2*AB*⋯O28^ii^	0.91	2.22	3.085 (5)	158
C26—H26*A*⋯Cl9	0.99	2.73	3.581 (4)	144

Symmetry codes: (i) 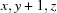; (ii) 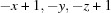.

**Table 3:** Experimental details

	(I)	(II)
Crystal data		
Chemical formula	[PtCl_2_(C_6_H_6_ClN)(C_13_H_16_O_4_)]·0.1H_2_O	[PtCl_2_(C_6_H_6_ClN)(C_14_H_18_O_4_)]
*M*_r_	631.61	643.84
Crystal system, space group	Monoclinic, *C*2/*c*	Triclinic, *P* 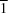
Temperature (K)	100	100
*a*, *b*, *c* (Å)	13.8322 (6), 15.0753 (4), 21.0367 (9)	9.9093 (3), 10.0102 (3), 11.1414 (4)
α, β, γ (°)	90, 106.683 (5), 90	97.302 (3), 99.706 (3), 92.572 (2)
*V* (Å^3^)	4202.0 (3)	1078.01 (6)
*Z*	8	2
Radiation type	Mo *K*α	Mo *K*α
μ (mm^−1^)	7.09	6.91
Crystal size (mm)	0.20 × 0.10 × 0.10	0.30 × 0.30 × 0.15

Data collection		
Diffractometer	Agilent SuperNova diffractometer (single source at offset, Eos detector)	Agilent SuperNova diffractometer (single source at offset, Eos detector)
Absorption correction	Multi-scan (*CrysAlis PRO*; Agilent, 2012 ▸)	Multi-scan (*CrysAlis PRO*; Agilent, 2012 ▸)
*T*_min_, *T*_max_	0.715, 1.000	0.547, 1.000
No. of measured, independent and observed [*I* > 2σ(*I*)] reflections	44052, 4295, 4033	22329, 4378, 4177
*R*_int_	0.032	0.044
(sin θ/λ)_max_ (Å^−1^)	0.625	0.625

Refinement		
*R*[*F* ^2^ > 2σ(*F* ^2^)], *wR*(*F* ^2^), *S*	0.032, 0.064, 1.22	0.026, 0.057, 1.20
No. of reflections	4295	4378
No. of parameters	315	292
No. of restraints	291	264
H-atom treatment	H-atom parameters constrained	H-atom parameters constrained
Δρ_max_, Δρ_min_ (e Å^−3^)	1.28, −0.92	0.97, −0.96

Computer programs: *CrysAlis PRO* (Agilent, 2012 ▸), *SHELXS97* (Sheldrick, 2008 ▸), *SHELXL2014* (Sheldrick, 2015 ▸), *olex2.solve* (Bourhis *et al.*, 2015 ▸) and *OLEX2* (Dolomanov *et al.*, 2009 ▸).

## supplementary crystallographic information

### (I) trans-Dichlorido(4-chloroaniline-κN){3-methoxy-4-methoxycarbonylmethoxy-1-[(2,3-η)-prop-2-en-1-yl]benzene}platinum(II) 0.1-hydrate Crystal data

**Table:**

[PtCl_2_(C_6_H_6_ClN)(C_13_H_16_O_4_)]·0.1H_2_O	*F*(000) = 2440.0
*M**_r_* = 631.61	*D*_x_ = 1.997 Mg m^−^^3^
Monoclinic, *C*2/*c*	Mo *K*α radiation, λ = 0.71073 Å
*a* = 13.8322 (6) Å	Cell parameters from 22209 reflections
*b* = 15.0753 (4) Å	θ = 3.3–28.6°
*c* = 21.0367 (9) Å	µ = 7.09 mm^−^^1^
β = 106.683 (5)°	*T* = 100 K
*V* = 4202.0 (3) Å^3^	Block, yellow
*Z* = 8	0.2 × 0.1 × 0.1 mm

### (I) trans-Dichlorido(4-chloroaniline-κN){3-methoxy-4-methoxycarbonylmethoxy-1-[(2,3-η)-prop-2-en-1-yl]benzene}platinum(II) 0.1-hydrate Data collection

**Table:**

Agilent SuperNova diffractometer (single source at offset, Eos detector)	4295 independent reflections
Radiation source: SuperNova (Mo) X-ray Source	4033 reflections with *I* > 2σ(*I*)
Mirror monochromator	*R*_int_ = 0.032
Detector resolution: 15.9631 pixels mm^-1^	θ_max_ = 26.4°, θ_min_ = 2.9°
ω scans	*h* = −17→17
Absorption correction: multi-scan (CrysAlis PRO; Agilent, 2012)	*k* = −18→18
*T*_min_ = 0.715, *T*_max_ = 1.000	*l* = −26→26
44052 measured reflections	

### (I) trans-Dichlorido(4-chloroaniline-κN){3-methoxy-4-methoxycarbonylmethoxy-1-[(2,3-η)-prop-2-en-1-yl]benzene}platinum(II) 0.1-hydrate Refinement

**Table:**

Refinement on *F*^2^	Primary atom site location: heavy-atom method
Least-squares matrix: full	Hydrogen site location: inferred from neighbouring sites
*R*[*F*^2^ > 2σ(*F*^2^)] = 0.032	H-atom parameters constrained
*wR*(*F*^2^) = 0.064	*w* = 1/[σ^2^(*F*_o_^2^) + 46.5817*P*] where *P* = (*F*_o_^2^ + 2*F*_c_^2^)/3
*S* = 1.22	(Δ/σ)_max_ < 0.001
4295 reflections	Δρ_max_ = 1.28 e Å^−^^3^
315 parameters	Δρ_min_ = −0.92 e Å^−^^3^
291 restraints	

### (I) trans-Dichlorido(4-chloroaniline-κN){3-methoxy-4-methoxycarbonylmethoxy-1-[(2,3-η)-prop-2-en-1-yl]benzene}platinum(II) 0.1-hydrate Special details

**Table:**

Geometry. All esds (except the esd in the dihedral angle between two l.s. planes) are estimated using the full covariance matrix. The cell esds are taken into account individually in the estimation of esds in distances, angles and torsion angles; correlations between esds in cell parameters are only used when they are defined by crystal symmetry. An approximate (isotropic) treatment of cell esds is used for estimating esds involving l.s. planes.

### (I) trans-Dichlorido(4-chloroaniline-κN){3-methoxy-4-methoxycarbonylmethoxy-1-[(2,3-η)-prop-2-en-1-yl]benzene}platinum(II) 0.1-hydrate Fractional atomic coordinates and isotropic or equivalent isotropic displacement parameters (Å^2^)

**Table:**

	*x*	*y*	*z*	*U*_iso_*/*U*_eq_	Occ. (<1)
Pt1A	0.75061 (14)	0.49179 (7)	0.82720 (10)	0.0213 (2)	0.679 (8)
Pt1B	0.7676 (3)	0.4904 (2)	0.8417 (2)	0.0349 (6)	0.321 (8)
N2	0.8940 (3)	0.5396 (3)	0.8235 (2)	0.0369 (10)	
H2AA	0.9427	0.4996	0.8432	0.044*	0.679 (8)
H2AB	0.8938	0.5459	0.7805	0.044*	0.679 (8)
H2BC	0.9465	0.5015	0.8389	0.044*	0.321 (8)
H2BD	0.8835	0.5469	0.7791	0.044*	0.321 (8)
C3	0.9164 (4)	0.6242 (3)	0.8573 (3)	0.0328 (11)	
C4	0.9600 (5)	0.6278 (4)	0.9245 (3)	0.0457 (14)	
H4	0.9792	0.5743	0.9487	0.055*	
C5	0.9763 (5)	0.7077 (4)	0.9572 (3)	0.0491 (15)	
H5	1.0047	0.7096	1.0040	0.059*	
C6	0.9509 (4)	0.7856 (4)	0.9213 (3)	0.0390 (12)	
C7	0.9087 (4)	0.7838 (4)	0.8539 (3)	0.0327 (11)	
H7	0.8915	0.8374	0.8295	0.039*	
C8	0.8913 (4)	0.7023 (3)	0.8218 (3)	0.0303 (11)	
H8	0.8621	0.7002	0.7751	0.036*	
Cl9	0.97029 (14)	0.88741 (11)	0.96197 (8)	0.0557 (4)	
Cl10	0.8612 (7)	0.4286 (5)	0.9395 (3)	0.0395 (16)	0.321 (8)
Cl11	0.6918 (4)	0.5297 (5)	0.7175 (2)	0.0425 (11)	0.679 (8)
Cl12	0.8205 (3)	0.4484 (2)	0.93519 (14)	0.0337 (6)	0.679 (8)
Cl13	0.6770 (6)	0.5272 (9)	0.7318 (4)	0.036 (2)	0.321 (8)
C14	0.6190 (7)	0.4194 (5)	0.8275 (4)	0.0265 (19)	0.679 (8)
H14A	0.6296	0.3818	0.8652	0.032*	0.679 (8)
H14B	0.6241	0.3962	0.7867	0.032*	0.679 (8)
C15	0.5959 (5)	0.5080 (4)	0.8322 (3)	0.0213 (15)	0.679 (8)
H15	0.5489	0.5324	0.7905	0.026*	0.679 (8)
C16	0.5856 (7)	0.5525 (4)	0.8945 (4)	0.0271 (18)	0.679 (8)
H16A	0.6411	0.5339	0.9335	0.032*	0.679 (8)
H16B	0.5205	0.5363	0.9022	0.032*	0.679 (8)
C17	0.6445 (18)	0.4012 (12)	0.8481 (11)	0.041 (5)	0.321 (8)
H17A	0.5949	0.3889	0.8073	0.050*	0.321 (8)
H17B	0.6967	0.3596	0.8662	0.050*	0.321 (8)
C18	0.6407 (13)	0.4798 (11)	0.8811 (10)	0.046 (4)	0.321 (8)
H18	0.6633	0.4711	0.9302	0.055*	0.321 (8)
C19	0.5657 (13)	0.5548 (7)	0.8620 (10)	0.042 (4)	0.321 (8)
H19A	0.5071	0.5376	0.8774	0.051*	0.321 (8)
H19B	0.5416	0.5552	0.8129	0.051*	0.321 (8)
C20	0.5902 (4)	0.6522 (4)	0.8837 (3)	0.0424 (13)	
C21	0.5064 (4)	0.7011 (3)	0.8465 (3)	0.0339 (11)	
H21	0.4456	0.6712	0.8242	0.041*	
C22	0.5118 (4)	0.7926 (3)	0.8422 (2)	0.0282 (10)	
C23	0.6008 (4)	0.8361 (3)	0.8764 (3)	0.0313 (11)	
C24	0.6828 (4)	0.7872 (4)	0.9122 (3)	0.0395 (12)	
H24	0.7435	0.8167	0.9352	0.047*	
C25	0.6774 (4)	0.6956 (4)	0.9150 (3)	0.0440 (13)	
H25	0.7349	0.6627	0.9389	0.053*	
O26	0.4355 (3)	0.8454 (2)	0.80566 (18)	0.0324 (8)	
C27	0.3460 (4)	0.8027 (4)	0.7673 (3)	0.0343 (12)	
H27A	0.3117	0.7750	0.7970	0.051*	
H27B	0.3635	0.7571	0.7393	0.051*	
H27C	0.3012	0.8465	0.7393	0.051*	
O28	0.5993 (3)	0.9267 (2)	0.86886 (19)	0.0372 (9)	
C29	0.6894 (5)	0.9716 (4)	0.9030 (3)	0.0446 (14)	
H29A	0.7479	0.9457	0.8914	0.054*	
H29B	0.7014	0.9667	0.9515	0.054*	
C30	0.6754 (5)	1.0688 (4)	0.8813 (3)	0.0480 (14)	
O31	0.6176 (4)	1.0965 (3)	0.8301 (3)	0.0582 (12)	
O32	0.7487 (5)	1.1132 (3)	0.9246 (2)	0.0740 (16)	
C33	0.7493 (9)	1.2074 (5)	0.9135 (5)	0.102 (3)	
H33A	0.7428	1.2188	0.8666	0.154*	
H33B	0.8128	1.2327	0.9410	0.154*	
H33C	0.6926	1.2349	0.9253	0.154*	
O34	1.0000	0.5000	0.7500	0.10 (2)	0.10 (1)

### (I) trans-Dichlorido(4-chloroaniline-κN){3-methoxy-4-methoxycarbonylmethoxy-1-[(2,3-η)-prop-2-en-1-yl]benzene}platinum(II) 0.1-hydrate Atomic displacement parameters (Å^2^)

**Table:**

	*U*^11^	*U*^22^	*U*^33^	*U*^12^	*U*^13^	*U*^23^
Pt1A	0.0216 (4)	0.0167 (3)	0.0252 (5)	−0.00062 (18)	0.0064 (4)	−0.0012 (2)
Pt1B	0.0269 (10)	0.0479 (9)	0.0288 (12)	−0.0027 (5)	0.0061 (9)	−0.0021 (6)
N2	0.028 (2)	0.029 (2)	0.054 (3)	0.0035 (17)	0.0125 (19)	0.0060 (19)
C3	0.022 (2)	0.033 (2)	0.043 (3)	−0.0006 (19)	0.008 (2)	0.006 (2)
C4	0.045 (3)	0.039 (3)	0.046 (3)	−0.014 (2)	0.001 (2)	0.013 (2)
C5	0.052 (4)	0.052 (3)	0.039 (3)	−0.020 (3)	0.005 (3)	0.009 (2)
C6	0.034 (3)	0.041 (3)	0.042 (3)	−0.018 (2)	0.011 (2)	0.001 (2)
C7	0.027 (3)	0.033 (2)	0.040 (3)	−0.003 (2)	0.012 (2)	0.008 (2)
C8	0.022 (2)	0.032 (2)	0.036 (3)	0.0006 (19)	0.008 (2)	0.0055 (19)
Cl9	0.0722 (11)	0.0515 (9)	0.0477 (9)	−0.0338 (8)	0.0242 (8)	−0.0125 (7)
Cl10	0.049 (4)	0.037 (3)	0.029 (2)	−0.003 (3)	0.005 (3)	0.008 (2)
Cl11	0.058 (3)	0.0403 (18)	0.0382 (14)	0.0123 (18)	0.0281 (13)	0.0128 (11)
Cl12	0.0287 (15)	0.0273 (13)	0.0359 (12)	0.0024 (10)	−0.0052 (11)	0.0095 (9)
Cl13	0.015 (2)	0.035 (3)	0.054 (5)	−0.005 (2)	0.004 (3)	0.016 (4)
C14	0.025 (4)	0.027 (4)	0.028 (4)	−0.001 (3)	0.008 (3)	0.001 (3)
C15	0.015 (3)	0.027 (3)	0.020 (3)	0.000 (2)	0.002 (2)	0.003 (2)
C16	0.036 (4)	0.029 (3)	0.018 (4)	0.005 (3)	0.011 (3)	0.007 (3)
C17	0.032 (8)	0.039 (7)	0.051 (10)	0.004 (5)	0.010 (7)	0.009 (6)
C18	0.042 (7)	0.041 (6)	0.058 (9)	0.002 (5)	0.019 (6)	0.010 (6)
C19	0.042 (8)	0.041 (5)	0.057 (11)	0.008 (4)	0.033 (7)	0.022 (5)
C20	0.041 (3)	0.035 (2)	0.061 (3)	0.007 (2)	0.030 (3)	0.013 (2)
C21	0.030 (2)	0.030 (2)	0.049 (3)	0.0014 (19)	0.022 (2)	0.001 (2)
C22	0.031 (2)	0.028 (2)	0.030 (2)	0.0048 (18)	0.0156 (19)	−0.0011 (18)
C23	0.034 (2)	0.033 (2)	0.028 (2)	0.0011 (19)	0.012 (2)	0.0007 (19)
C24	0.037 (3)	0.047 (3)	0.034 (3)	0.002 (2)	0.010 (2)	0.006 (2)
C25	0.035 (3)	0.047 (3)	0.052 (3)	0.009 (2)	0.016 (3)	0.017 (2)
O26	0.0293 (18)	0.0261 (17)	0.040 (2)	0.0027 (14)	0.0081 (15)	−0.0015 (15)
C27	0.030 (3)	0.038 (3)	0.035 (3)	0.002 (2)	0.010 (2)	−0.004 (2)
O28	0.037 (2)	0.0302 (18)	0.041 (2)	−0.0044 (15)	0.0062 (17)	−0.0065 (15)
C29	0.046 (3)	0.049 (3)	0.039 (3)	−0.015 (2)	0.011 (3)	−0.004 (2)
C30	0.061 (4)	0.042 (3)	0.050 (3)	−0.017 (3)	0.029 (3)	−0.005 (2)
O31	0.052 (3)	0.051 (3)	0.076 (3)	0.001 (2)	0.026 (2)	0.007 (2)
O32	0.110 (4)	0.066 (3)	0.050 (3)	−0.052 (3)	0.030 (3)	−0.011 (2)
C33	0.171 (10)	0.062 (4)	0.100 (7)	−0.063 (5)	0.081 (7)	−0.018 (4)
O34	0.06 (3)	0.21 (6)	0.04 (2)	0.000	0.015 (18)	0.000

### (I) trans-Dichlorido(4-chloroaniline-κN){3-methoxy-4-methoxycarbonylmethoxy-1-[(2,3-η)-prop-2-en-1-yl]benzene}platinum(II) 0.1-hydrate Geometric parameters (Å, º)

**Table:**

Pt1A—N2	2.132 (5)	C17—H17A	0.9500
Pt1A—Cl11	2.288 (4)	C17—H17B	0.9500
Pt1A—Cl12	2.294 (3)	C17—C18	1.381 (12)
Pt1A—C14	2.124 (9)	C18—H18	1.0000
Pt1A—C15	2.187 (6)	C18—C19	1.508 (11)
Pt1B—N2	2.033 (6)	C19—H19A	0.9900
Pt1B—Cl10	2.291 (5)	C19—H19B	0.9900
Pt1B—Cl13	2.357 (7)	C19—C20	1.546 (11)
Pt1B—C17	2.20 (2)	C20—C21	1.407 (8)
Pt1B—C18	2.151 (18)	C20—C25	1.364 (9)
N2—H2AA	0.9100	C21—H21	0.9500
N2—H2AB	0.9100	C21—C22	1.386 (7)
N2—H2BC	0.9100	C22—C23	1.398 (7)
N2—H2BD	0.9100	C22—O26	1.369 (6)
N2—C3	1.450 (7)	C23—C24	1.381 (8)
C3—C4	1.370 (8)	C23—O28	1.374 (6)
C3—C8	1.384 (7)	C24—H24	0.9500
C4—H4	0.9500	C24—C25	1.385 (8)
C4—C5	1.374 (9)	C25—H25	0.9500
C5—H5	0.9500	O26—C27	1.422 (6)
C5—C6	1.386 (8)	C27—H27A	0.9800
C6—C7	1.370 (8)	C27—H27B	0.9800
C6—Cl9	1.741 (6)	C27—H27C	0.9800
C7—H7	0.9500	O28—C29	1.419 (7)
C7—C8	1.389 (7)	C29—H29A	0.9900
C8—H8	0.9500	C29—H29B	0.9900
C14—H14A	0.9500	C29—C30	1.530 (9)
C14—H14B	0.9500	C30—O31	1.218 (8)
C14—C15	1.383 (9)	C30—O32	1.332 (8)
C15—H15	1.0000	O32—C33	1.440 (9)
C15—C16	1.514 (8)	C33—H33A	0.9800
C16—H16A	0.9900	C33—H33B	0.9800
C16—H16B	0.9900	C33—H33C	0.9800
C16—C20	1.523 (8)		
			
N2—Pt1A—Cl11	86.8 (2)	H16A—C16—H16B	108.6
N2—Pt1A—Cl12	90.03 (16)	C20—C16—H16A	110.4
N2—Pt1A—C15	153.8 (2)	C20—C16—H16B	110.4
Cl11—Pt1A—Cl12	175.35 (16)	Pt1B—C17—H17A	115.8
C14—Pt1A—N2	168.7 (2)	Pt1B—C17—H17B	85.2
C14—Pt1A—Cl11	94.3 (3)	H17A—C17—H17B	120.0
C14—Pt1A—Cl12	88.2 (3)	C18—C17—Pt1B	69.4 (12)
C14—Pt1A—C15	37.4 (3)	C18—C17—H17A	120.0
C15—Pt1A—Cl11	87.2 (2)	C18—C17—H17B	120.0
C15—Pt1A—Cl12	97.1 (2)	Pt1B—C18—H18	111.2
N2—Pt1B—Cl10	91.2 (2)	C17—C18—Pt1B	73.6 (13)
N2—Pt1B—Cl13	88.5 (3)	C17—C18—H18	111.2
N2—Pt1B—C17	162.7 (5)	C17—C18—C19	129.1 (17)
N2—Pt1B—C18	160.0 (5)	C19—C18—Pt1B	114.6 (12)
Cl10—Pt1B—Cl13	168.6 (4)	C19—C18—H18	111.2
C17—Pt1B—Cl10	86.8 (6)	C18—C19—H19A	106.4
C17—Pt1B—Cl13	90.1 (6)	C18—C19—H19B	106.4
C18—Pt1B—Cl10	86.4 (6)	C18—C19—C20	123.8 (13)
C18—Pt1B—Cl13	97.6 (6)	H19A—C19—H19B	106.4
C18—Pt1B—C17	36.9 (4)	C20—C19—H19A	106.4
Pt1A—N2—H2AA	109.5	C20—C19—H19B	106.4
Pt1A—N2—H2AB	109.5	C21—C20—C16	122.4 (6)
Pt1B—N2—H2BC	110.2	C21—C20—C19	104.9 (8)
Pt1B—N2—H2BD	110.2	C25—C20—C16	118.1 (6)
H2AA—N2—H2AB	108.1	C25—C20—C19	133.4 (8)
H2BC—N2—H2BD	108.5	C25—C20—C21	119.3 (5)
C3—N2—Pt1A	110.5 (3)	C20—C21—H21	119.8
C3—N2—Pt1B	107.7 (3)	C22—C21—C20	120.4 (5)
C3—N2—H2AA	109.5	C22—C21—H21	119.8
C3—N2—H2AB	109.5	C21—C22—C23	119.3 (5)
C3—N2—H2BC	110.2	O26—C22—C21	124.6 (5)
C3—N2—H2BD	110.2	O26—C22—C23	116.1 (4)
C4—C3—N2	120.5 (5)	C24—C23—C22	119.7 (5)
C4—C3—C8	119.5 (5)	O28—C23—C22	115.1 (5)
C8—C3—N2	120.0 (5)	O28—C23—C24	125.2 (5)
C3—C4—H4	119.6	C23—C24—H24	119.7
C3—C4—C5	120.8 (6)	C23—C24—C25	120.6 (6)
C5—C4—H4	119.6	C25—C24—H24	119.7
C4—C5—H5	120.3	C20—C25—C24	120.7 (6)
C4—C5—C6	119.3 (6)	C20—C25—H25	119.7
C6—C5—H5	120.3	C24—C25—H25	119.7
C5—C6—Cl9	119.9 (5)	C22—O26—C27	117.5 (4)
C7—C6—C5	121.0 (6)	O26—C27—H27A	109.5
C7—C6—Cl9	119.1 (4)	O26—C27—H27B	109.5
C6—C7—H7	120.6	O26—C27—H27C	109.5
C6—C7—C8	118.9 (5)	H27A—C27—H27B	109.5
C8—C7—H7	120.6	H27A—C27—H27C	109.5
C3—C8—C7	120.5 (5)	H27B—C27—H27C	109.5
C3—C8—H8	119.7	C23—O28—C29	115.7 (4)
C7—C8—H8	119.7	O28—C29—H29A	110.4
Pt1A—C14—H14A	112.4	O28—C29—H29B	110.4
Pt1A—C14—H14B	84.2	O28—C29—C30	106.8 (5)
H14A—C14—H14B	120.0	H29A—C29—H29B	108.6
C15—C14—Pt1A	73.8 (4)	C30—C29—H29A	110.4
C15—C14—H14A	120.0	C30—C29—H29B	110.4
C15—C14—H14B	120.0	O31—C30—C29	125.9 (6)
Pt1A—C15—H15	113.4	O31—C30—O32	127.7 (6)
C14—C15—Pt1A	68.9 (5)	O32—C30—C29	105.7 (6)
C14—C15—H15	113.4	C30—O32—C33	115.0 (7)
C14—C15—C16	124.8 (6)	O32—C33—H33A	109.5
C16—C15—Pt1A	115.3 (5)	O32—C33—H33B	109.5
C16—C15—H15	113.4	O32—C33—H33C	109.5
C15—C16—H16A	110.4	H33A—C33—H33B	109.5
C15—C16—H16B	110.4	H33A—C33—H33C	109.5
C15—C16—C20	106.8 (5)	H33B—C33—H33C	109.5
			
Pt1A—N2—C3—C4	85.5 (5)	C18—C19—C20—C21	170.4 (16)
Pt1A—N2—C3—C8	−92.9 (5)	C18—C19—C20—C25	9 (3)
Pt1A—C14—C15—C16	−106.8 (7)	C19—C20—C21—C22	−165.6 (9)
Pt1A—C15—C16—C20	83.1 (6)	C19—C20—C25—C24	161.9 (12)
Pt1B—N2—C3—C4	76.9 (6)	C20—C21—C22—C23	−1.4 (8)
Pt1B—N2—C3—C8	−101.5 (5)	C20—C21—C22—O26	177.9 (5)
Pt1B—C17—C18—C19	109 (2)	C21—C20—C25—C24	2.1 (9)
Pt1B—C18—C19—C20	−65 (2)	C21—C22—C23—C24	1.9 (7)
N2—C3—C4—C5	−176.4 (5)	C21—C22—C23—O28	179.7 (4)
N2—C3—C8—C7	177.4 (5)	C21—C22—O26—C27	−2.8 (7)
C3—C4—C5—C6	−1.9 (10)	C22—C23—C24—C25	−0.5 (8)
C4—C3—C8—C7	−1.0 (8)	C22—C23—O28—C29	−179.6 (5)
C4—C5—C6—C7	0.9 (9)	C23—C22—O26—C27	176.5 (4)
C4—C5—C6—Cl9	179.6 (5)	C23—C24—C25—C20	−1.6 (9)
C5—C6—C7—C8	0.1 (8)	C23—O28—C29—C30	173.5 (4)
C6—C7—C8—C3	0.0 (8)	C24—C23—O28—C29	−2.0 (8)
C8—C3—C4—C5	2.0 (9)	C25—C20—C21—C22	−0.6 (8)
Cl9—C6—C7—C8	−178.7 (4)	O26—C22—C23—C24	−177.4 (5)
C14—C15—C16—C20	164.0 (7)	O26—C22—C23—O28	0.3 (6)
C15—C16—C20—C21	80.3 (8)	O28—C23—C24—C25	−178.0 (5)
C15—C16—C20—C25	−104.0 (7)	O28—C29—C30—O31	−23.3 (9)
C16—C20—C21—C22	175.0 (5)	O28—C29—C30—O32	165.6 (5)
C16—C20—C25—C24	−173.7 (6)	C29—C30—O32—C33	−179.7 (6)
C17—C18—C19—C20	−153 (2)	O31—C30—O32—C33	9.4 (11)

### (I) trans-Dichlorido(4-chloroaniline-κN){3-methoxy-4-methoxycarbonylmethoxy-1-[(2,3-η)-prop-2-en-1-yl]benzene}platinum(II) 0.1-hydrate Hydrogen-bond geometry (Å, º)

Cg1 is the centroid of the C20–C25 ring.

**Table:**

*D*—H···*A*	*D*—H	H···*A*	*D*···*A*	*D*—H···*A*
N2—H2*AA*···O26^i^	0.91	2.45	3.027 (5)	122
N2—H2*AA*···O28^i^	0.91	2.35	3.212 (6)	158
N2—H2*AB*···O31^ii^	0.91	2.41	3.302 (3)	167
C16—H16*A*···Cl12	0.99	2.79	3.487 (10)	128
C16—H16*B*···Cl9^iii^	0.99	2.76	3.472 (8)	129
C21—H21···Cl11^iv^	0.95	2.82	3.726 (8)	159
C29—H29*B*···Cl12^v^	0.99	2.80	3.651 (7)	145
C27—H27*B*···*Cg*1^iv^	0.98	2.72	3.523 (6)	139

Symmetry codes: (i) *x*+1/2, *y*−1/2, *z*; (ii) −*x*+3/2, *y*−1/2, −*z*+3/2; (iii) *x*−1/2, *y*−1/2, *z*; (iv) −*x*+1, *y*, −*z*+3/2; (v) −*x*+3/2, −*y*+3/2, −*z*+2.

### (II) trans-Dichlorido(4-chloroaniline-κN){4-ethoxycarbonylmethoxy-3-methoxy-1-[(2,3-η)-prop-2-en-1-yl]benzene}platinum(II) Crystal data

**Table:**

[PtCl_2_(C_6_H_6_ClN)(C_14_H_18_O_4_)]	*Z* = 2
*M**_r_* = 643.84	*F*(000) = 624
Triclinic, *P*1	*D*_x_ = 1.984 Mg m^−^^3^
*a* = 9.9093 (3) Å	Mo *K*α radiation, λ = 0.71073 Å
*b* = 10.0102 (3) Å	Cell parameters from 10730 reflections
*c* = 11.1414 (4) Å	θ = 3.2–29.0°
α = 97.302 (3)°	µ = 6.91 mm^−^^1^
β = 99.706 (3)°	*T* = 100 K
γ = 92.572 (2)°	Block, yellow
*V* = 1078.01 (6) Å^3^	0.3 × 0.3 × 0.15 mm

### (II) trans-Dichlorido(4-chloroaniline-κN){4-ethoxycarbonylmethoxy-3-methoxy-1-[(2,3-η)-prop-2-en-1-yl]benzene}platinum(II) Data collection

**Table:**

Agilent SuperNova diffractometer (single source at offset, Eos detector)	4378 independent reflections
Radiation source: SuperNova (Mo) X-ray Source	4177 reflections with *I* > 2σ(*I*)
Mirror monochromator	*R*_int_ = 0.044
Detector resolution: 15.9631 pixels mm^-1^	θ_max_ = 26.4°, θ_min_ = 2.8°
ω scans	*h* = −12→12
Absorption correction: multi-scan (CrysAlis PRO; Agilent, 2012)	*k* = −12→12
*T*_min_ = 0.547, *T*_max_ = 1.000	*l* = −13→13
22329 measured reflections	

### (II) trans-Dichlorido(4-chloroaniline-κN){4-ethoxycarbonylmethoxy-3-methoxy-1-[(2,3-η)-prop-2-en-1-yl]benzene}platinum(II) Refinement

**Table:**

Refinement on *F*^2^	Primary atom site location: iterative
Least-squares matrix: full	Hydrogen site location: inferred from neighbouring sites
*R*[*F*^2^ > 2σ(*F*^2^)] = 0.026	H-atom parameters constrained
*wR*(*F*^2^) = 0.057	*w* = 1/[σ^2^(*F*_o_^2^) + (0.0056*P*)^2^ + 3.1214*P*] where *P* = (*F*_o_^2^ + 2*F*_c_^2^)/3
*S* = 1.20	(Δ/σ)_max_ < 0.001
4378 reflections	Δρ_max_ = 0.97 e Å^−^^3^
292 parameters	Δρ_min_ = −0.96 e Å^−^^3^
264 restraints	

### (II) trans-Dichlorido(4-chloroaniline-κN){4-ethoxycarbonylmethoxy-3-methoxy-1-[(2,3-η)-prop-2-en-1-yl]benzene}platinum(II) Special details

**Table:**

Geometry. All esds (except the esd in the dihedral angle between two l.s. planes) are estimated using the full covariance matrix. The cell esds are taken into account individually in the estimation of esds in distances, angles and torsion angles; correlations between esds in cell parameters are only used when they are defined by crystal symmetry. An approximate (isotropic) treatment of cell esds is used for estimating esds involving l.s. planes.

### (II) trans-Dichlorido(4-chloroaniline-κN){4-ethoxycarbonylmethoxy-3-methoxy-1-[(2,3-η)-prop-2-en-1-yl]benzene}platinum(II) Fractional atomic coordinates and isotropic or equivalent isotropic displacement parameters (Å^2^)

**Table:**

	*x*	*y*	*z*	*U*_iso_*/*U*_eq_	Occ. (<1)
Pt1A	0.69549 (11)	0.29586 (10)	0.25083 (7)	0.01218 (14)	0.872 (6)
Pt1B	0.6572 (11)	0.2618 (9)	0.2299 (6)	0.0263 (11)	0.128 (6)
N2	0.6352 (4)	0.3773 (4)	0.4142 (3)	0.0175 (7)	
H2AA	0.6687	0.4649	0.4346	0.021*	0.872 (6)
H2AB	0.5420	0.3754	0.4033	0.021*	0.872 (6)
H2BC	0.6834	0.4588	0.4258	0.021*	0.128 (6)
H2BD	0.5454	0.3921	0.4147	0.021*	0.128 (6)
C3	0.6861 (4)	0.3007 (4)	0.5131 (4)	0.0172 (9)	
C4	0.8223 (4)	0.3217 (4)	0.5704 (4)	0.0191 (9)	
H4	0.8815	0.3854	0.5446	0.023*	
C5	0.8722 (4)	0.2504 (4)	0.6650 (4)	0.0194 (9)	
H5	0.9653	0.2646	0.7049	0.023*	
C6	0.7836 (4)	0.1577 (4)	0.7002 (4)	0.0182 (9)	
C7	0.6483 (4)	0.1340 (4)	0.6433 (4)	0.0191 (9)	
H7	0.5897	0.0692	0.6685	0.023*	
C8	0.5994 (4)	0.2062 (4)	0.5490 (4)	0.0199 (9)	
H8	0.5065	0.1912	0.5088	0.024*	
Cl9	0.84620 (12)	0.06618 (11)	0.81957 (10)	0.0252 (2)	
Cl10	0.88980 (15)	0.4404 (2)	0.30141 (16)	0.0170 (4)	0.872 (6)
Cl11	0.4916 (2)	0.1647 (2)	0.20164 (18)	0.0173 (4)	0.872 (6)
Cl12	0.8689 (13)	0.377 (2)	0.2675 (13)	0.037 (3)	0.128 (6)
Cl13	0.4387 (19)	0.1586 (18)	0.1984 (16)	0.034 (3)	0.128 (6)
C14	0.7274 (5)	0.2580 (5)	0.0645 (4)	0.0236 (10)	
H14A	0.6473	0.2280	0.0057	0.028*	
H14B	0.7579	0.3509	0.0794	0.028*	
C15	0.8002 (5)	0.1655 (5)	0.1289 (4)	0.0242 (10)	
H15	0.9021	0.1839	0.1437	0.029*	0.872 (6)
H15A	0.9000	0.1894	0.1608	0.029*	0.128 (6)
C16	0.7539 (5)	0.0195 (4)	0.1067 (4)	0.0245 (10)	
H16A	0.8108	−0.0272	0.0517	0.029*	
H16B	0.6582	0.0106	0.0616	0.029*	
C17	0.7586 (5)	−0.0551 (4)	0.2180 (4)	0.0186 (9)	
C18	0.6816 (4)	−0.1797 (4)	0.1979 (4)	0.0187 (9)	
H18	0.6308	−0.2117	0.1184	0.022*	
C19	0.6787 (4)	−0.2565 (4)	0.2923 (4)	0.0161 (8)	
C20	0.7528 (4)	−0.2097 (4)	0.4101 (4)	0.0143 (8)	
C21	0.8282 (4)	−0.0865 (4)	0.4312 (4)	0.0174 (9)	
H21	0.8780	−0.0536	0.5108	0.021*	
C22	0.8303 (4)	−0.0106 (4)	0.3335 (4)	0.0196 (9)	
H22	0.8827	0.0736	0.3480	0.023*	
O23	0.6053 (3)	−0.3770 (3)	0.2818 (3)	0.0183 (6)	
C24	0.5250 (5)	−0.4260 (4)	0.1644 (4)	0.0241 (10)	
H24A	0.5845	−0.4341	0.1023	0.036*	
H24B	0.4549	−0.3629	0.1430	0.036*	
H24C	0.4805	−0.5147	0.1672	0.036*	
O25	0.7390 (3)	−0.2930 (3)	0.4975 (3)	0.0168 (6)	
C26	0.8067 (4)	−0.2464 (4)	0.6172 (4)	0.0183 (9)	
H26A	0.7842	−0.1526	0.6418	0.022*	
H26B	0.9072	−0.2473	0.6214	0.022*	
C27	0.7604 (4)	−0.3380 (4)	0.7025 (4)	0.0157 (8)	
O28	0.6689 (3)	−0.4258 (3)	0.6725 (3)	0.0176 (6)	
O29	0.8362 (3)	−0.3074 (3)	0.8142 (3)	0.0180 (6)	
C30	0.8025 (5)	−0.3861 (5)	0.9070 (4)	0.0212 (10)	
H30A	0.8025	−0.4837	0.8776	0.025*	
H30B	0.7103	−0.3668	0.9249	0.025*	
C31	0.9091 (4)	−0.3475 (4)	1.0205 (4)	0.0200 (9)	
H31A	0.9993	−0.3714	1.0028	0.030*	
H31B	0.8865	−0.3960	1.0864	0.030*	
H31C	0.9112	−0.2501	1.0465	0.030*	

### (II) trans-Dichlorido(4-chloroaniline-κN){4-ethoxycarbonylmethoxy-3-methoxy-1-[(2,3-η)-prop-2-en-1-yl]benzene}platinum(II) Atomic displacement parameters (Å^2^)

**Table:**

	*U*^11^	*U*^22^	*U*^33^	*U*^12^	*U*^13^	*U*^23^
Pt1A	0.0123 (3)	0.0118 (2)	0.01192 (18)	−0.00266 (15)	0.00058 (15)	0.00299 (14)
Pt1B	0.030 (2)	0.030 (2)	0.0207 (16)	−0.0011 (19)	0.0073 (16)	0.0081 (15)
N2	0.0171 (17)	0.0194 (18)	0.0155 (19)	−0.0007 (14)	0.0029 (14)	0.0010 (14)
C3	0.020 (2)	0.017 (2)	0.015 (2)	0.0008 (16)	0.0050 (16)	0.0017 (16)
C4	0.021 (2)	0.020 (2)	0.016 (2)	−0.0049 (17)	0.0044 (17)	0.0020 (17)
C5	0.018 (2)	0.024 (2)	0.015 (2)	−0.0034 (17)	0.0022 (17)	0.0002 (18)
C6	0.025 (2)	0.018 (2)	0.012 (2)	0.0003 (16)	0.0033 (17)	0.0014 (17)
C7	0.022 (2)	0.017 (2)	0.019 (2)	−0.0041 (16)	0.0076 (17)	0.0024 (17)
C8	0.0146 (19)	0.026 (2)	0.019 (2)	−0.0033 (17)	0.0030 (17)	0.0048 (18)
Cl9	0.0379 (6)	0.0240 (6)	0.0123 (6)	−0.0029 (5)	0.0008 (5)	0.0041 (4)
Cl10	0.0148 (6)	0.0174 (8)	0.0176 (8)	−0.0065 (5)	0.0010 (5)	0.0032 (6)
Cl11	0.0124 (8)	0.0174 (7)	0.0201 (8)	−0.0054 (7)	0.0009 (7)	−0.0005 (5)
Cl12	0.034 (5)	0.049 (8)	0.026 (6)	−0.013 (5)	0.010 (4)	0.003 (5)
Cl13	0.033 (7)	0.036 (6)	0.034 (7)	−0.002 (6)	0.011 (6)	0.002 (5)
C14	0.030 (2)	0.025 (2)	0.017 (2)	0.0025 (19)	0.0043 (18)	0.0060 (18)
C15	0.028 (2)	0.025 (2)	0.022 (2)	−0.0012 (18)	0.0084 (19)	0.0056 (18)
C16	0.040 (3)	0.020 (2)	0.014 (2)	−0.0026 (19)	0.008 (2)	0.0022 (17)
C17	0.031 (2)	0.0151 (19)	0.012 (2)	0.0047 (17)	0.0083 (17)	0.0035 (16)
C18	0.027 (2)	0.018 (2)	0.011 (2)	0.0009 (17)	0.0024 (17)	0.0029 (16)
C19	0.018 (2)	0.0155 (19)	0.015 (2)	0.0009 (15)	0.0048 (16)	0.0033 (15)
C20	0.0158 (19)	0.0154 (19)	0.013 (2)	0.0009 (15)	0.0025 (15)	0.0052 (15)
C21	0.019 (2)	0.019 (2)	0.013 (2)	−0.0031 (16)	0.0016 (16)	0.0022 (16)
C22	0.024 (2)	0.015 (2)	0.020 (2)	−0.0035 (17)	0.0058 (17)	0.0041 (17)
O23	0.0226 (15)	0.0182 (15)	0.0127 (16)	−0.0043 (12)	−0.0018 (12)	0.0046 (12)
C24	0.034 (3)	0.020 (2)	0.016 (2)	−0.0050 (19)	−0.0040 (19)	0.0067 (18)
O25	0.0236 (15)	0.0160 (14)	0.0098 (15)	−0.0047 (12)	−0.0009 (12)	0.0054 (12)
C26	0.019 (2)	0.019 (2)	0.015 (2)	−0.0050 (17)	−0.0003 (16)	0.0048 (17)
C27	0.0174 (19)	0.016 (2)	0.014 (2)	0.0026 (16)	0.0029 (15)	0.0020 (16)
O28	0.0221 (15)	0.0177 (15)	0.0112 (16)	−0.0046 (12)	0.0005 (12)	0.0009 (12)
O29	0.0186 (15)	0.0225 (16)	0.0123 (15)	−0.0057 (12)	−0.0003 (12)	0.0063 (12)
C30	0.027 (2)	0.024 (2)	0.013 (2)	−0.0056 (18)	0.0030 (18)	0.0045 (18)
C31	0.025 (2)	0.023 (2)	0.012 (2)	−0.0006 (18)	0.0004 (17)	0.0040 (18)

### (II) trans-Dichlorido(4-chloroaniline-κN){4-ethoxycarbonylmethoxy-3-methoxy-1-[(2,3-η)-prop-2-en-1-yl]benzene}platinum(II) Geometric parameters (Å, º)

**Table:**

Pt1A—N2	2.089 (4)	C16—H16A	0.9900
Pt1A—Cl10	2.3011 (14)	C16—H16B	0.9900
Pt1A—Cl11	2.3050 (15)	C16—C17	1.522 (6)
Pt1A—C14	2.142 (5)	C17—C18	1.406 (6)
Pt1A—C15	2.184 (5)	C17—C22	1.371 (6)
Pt1B—N2	2.273 (8)	C18—H18	0.9500
Pt1B—Cl12	2.297 (12)	C18—C19	1.382 (6)
Pt1B—Cl13	2.309 (14)	C19—C20	1.403 (6)
Pt1B—C14	2.073 (6)	C19—O23	1.362 (5)
Pt1B—C15	2.142 (6)	C20—C21	1.385 (6)
N2—H2AA	0.9100	C20—O25	1.380 (5)
N2—H2AB	0.9100	C21—H21	0.9500
N2—H2BC	0.9100	C21—C22	1.406 (6)
N2—H2BD	0.9100	C22—H22	0.9500
N2—C3	1.455 (5)	O23—C24	1.427 (5)
C3—C4	1.387 (6)	C24—H24A	0.9800
C3—C8	1.390 (6)	C24—H24B	0.9800
C4—H4	0.9500	C24—H24C	0.9800
C4—C5	1.383 (6)	O25—C26	1.399 (5)
C5—H5	0.9500	C26—H26A	0.9900
C5—C6	1.387 (6)	C26—H26B	0.9900
C6—C7	1.379 (6)	C26—C27	1.512 (5)
C6—Cl9	1.754 (4)	C27—O28	1.207 (5)
C7—H7	0.9500	C27—O29	1.332 (5)
C7—C8	1.384 (6)	O29—C30	1.449 (5)
C8—H8	0.9500	C30—H30A	0.9900
C14—H14A	0.9500	C30—H30B	0.9900
C14—H14B	0.9500	C30—C31	1.501 (6)
C14—C15	1.396 (6)	C31—H31A	0.9800
C15—H15	1.0000	C31—H31B	0.9800
C15—H15A	1.0000	C31—H31C	0.9800
C15—C16	1.489 (6)		
			
N2—Pt1A—Cl10	88.86 (10)	C14—C15—H15	114.2
N2—Pt1A—Cl11	88.77 (11)	C14—C15—H15A	117.2
N2—Pt1A—C14	163.99 (15)	C14—C15—C16	120.7 (4)
N2—Pt1A—C15	158.31 (16)	C16—C15—Pt1A	116.5 (3)
Cl10—Pt1A—Cl11	175.82 (7)	C16—C15—Pt1B	105.3 (4)
C14—Pt1A—Cl10	90.53 (14)	C16—C15—H15	114.2
C14—Pt1A—Cl11	90.80 (15)	C16—C15—H15A	117.2
C14—Pt1A—C15	37.62 (17)	C15—C16—H16A	107.9
C15—Pt1A—Cl10	89.13 (14)	C15—C16—H16B	107.9
C15—Pt1A—Cl11	94.33 (15)	C15—C16—C17	117.8 (4)
N2—Pt1B—Cl12	83.8 (4)	H16A—C16—H16B	107.2
N2—Pt1B—Cl13	93.1 (5)	C17—C16—H16A	107.9
Cl12—Pt1B—Cl13	176.1 (6)	C17—C16—H16B	107.9
C14—Pt1B—N2	149.1 (6)	C18—C17—C16	115.9 (4)
C14—Pt1B—Cl12	71.8 (5)	C22—C17—C16	125.5 (4)
C14—Pt1B—Cl13	110.3 (7)	C22—C17—C18	118.6 (4)
C14—Pt1B—C15	38.62 (18)	C17—C18—H18	119.6
C15—Pt1B—N2	143.8 (6)	C19—C18—C17	120.8 (4)
C15—Pt1B—Cl12	67.1 (6)	C19—C18—H18	119.6
C15—Pt1B—Cl13	116.7 (7)	C18—C19—C20	119.9 (4)
Pt1A—N2—H2AA	109.6	O23—C19—C18	124.8 (4)
Pt1A—N2—H2AB	109.6	O23—C19—C20	115.2 (3)
Pt1B—N2—H2BC	109.6	C21—C20—C19	119.7 (4)
Pt1B—N2—H2BD	109.6	O25—C20—C19	114.7 (3)
H2AA—N2—H2AB	108.1	O25—C20—C21	125.6 (4)
H2BC—N2—H2BD	108.2	C20—C21—H21	120.3
C3—N2—Pt1A	110.4 (3)	C20—C21—C22	119.4 (4)
C3—N2—Pt1B	110.1 (3)	C22—C21—H21	120.3
C3—N2—H2AA	109.6	C17—C22—C21	121.5 (4)
C3—N2—H2AB	109.6	C17—C22—H22	119.2
C3—N2—H2BC	109.6	C21—C22—H22	119.2
C3—N2—H2BD	109.6	C19—O23—C24	117.0 (3)
C4—C3—N2	119.5 (4)	O23—C24—H24A	109.5
C4—C3—C8	120.2 (4)	O23—C24—H24B	109.5
C8—C3—N2	120.4 (4)	O23—C24—H24C	109.5
C3—C4—H4	119.9	H24A—C24—H24B	109.5
C5—C4—C3	120.3 (4)	H24A—C24—H24C	109.5
C5—C4—H4	119.9	H24B—C24—H24C	109.5
C4—C5—H5	120.7	C20—O25—C26	116.1 (3)
C4—C5—C6	118.6 (4)	O25—C26—H26A	110.0
C6—C5—H5	120.7	O25—C26—H26B	110.0
C5—C6—Cl9	118.9 (3)	O25—C26—C27	108.3 (3)
C7—C6—C5	122.1 (4)	H26A—C26—H26B	108.4
C7—C6—Cl9	119.0 (3)	C27—C26—H26A	110.0
C6—C7—H7	120.6	C27—C26—H26B	110.0
C6—C7—C8	118.7 (4)	O28—C27—C26	124.6 (4)
C8—C7—H7	120.6	O28—C27—O29	125.8 (4)
C3—C8—H8	119.9	O29—C27—C26	109.7 (3)
C7—C8—C3	120.1 (4)	C27—O29—C30	116.1 (3)
C7—C8—H8	119.9	O29—C30—H30A	110.2
Pt1A—C14—H14A	115.0	O29—C30—H30B	110.2
Pt1A—C14—H14B	82.6	O29—C30—C31	107.6 (3)
H14A—C14—H14B	120.0	H30A—C30—H30B	108.5
C15—C14—Pt1A	72.9 (3)	C31—C30—H30A	110.2
C15—C14—Pt1B	73.4 (3)	C31—C30—H30B	110.2
C15—C14—H14A	120.0	C30—C31—H31A	109.5
C15—C14—H14B	120.0	C30—C31—H31B	109.5
Pt1A—C15—H15	114.2	C30—C31—H31C	109.5
Pt1B—C15—H15A	117.2	H31A—C31—H31B	109.5
C14—C15—Pt1A	69.5 (3)	H31A—C31—H31C	109.5
C14—C15—Pt1B	68.0 (3)	H31B—C31—H31C	109.5
			
Pt1A—N2—C3—C4	78.3 (4)	C16—C17—C22—C21	179.8 (4)
Pt1A—N2—C3—C8	−101.0 (4)	C17—C18—C19—C20	0.3 (6)
Pt1A—C14—C15—C16	109.4 (4)	C17—C18—C19—O23	178.8 (4)
Pt1A—C15—C16—C17	−56.7 (5)	C18—C17—C22—C21	0.0 (7)
Pt1B—N2—C3—C4	91.5 (5)	C18—C19—C20—C21	0.2 (6)
Pt1B—N2—C3—C8	−87.8 (5)	C18—C19—C20—O25	178.3 (4)
Pt1B—C14—C15—C16	95.3 (6)	C18—C19—O23—C24	−0.5 (6)
Pt1B—C15—C16—C17	−64.4 (5)	C19—C20—C21—C22	−0.6 (6)
N2—C3—C4—C5	179.7 (4)	C19—C20—O25—C26	−177.2 (3)
N2—C3—C8—C7	−179.8 (4)	C20—C19—O23—C24	178.1 (4)
C3—C4—C5—C6	0.3 (7)	C20—C21—C22—C17	0.5 (7)
C4—C3—C8—C7	0.9 (7)	C20—O25—C26—C27	170.4 (3)
C4—C5—C6—C7	0.6 (7)	C21—C20—O25—C26	0.7 (6)
C4—C5—C6—Cl9	179.7 (3)	C22—C17—C18—C19	−0.4 (6)
C5—C6—C7—C8	−0.7 (7)	O23—C19—C20—C21	−178.5 (4)
C6—C7—C8—C3	0.0 (7)	O23—C19—C20—O25	−0.4 (5)
C8—C3—C4—C5	−1.0 (7)	O25—C20—C21—C22	−178.4 (4)
Cl9—C6—C7—C8	−179.8 (3)	O25—C26—C27—O28	−7.9 (6)
C14—C15—C16—C17	−137.6 (5)	O25—C26—C27—O29	171.8 (3)
C15—C16—C17—C18	163.5 (4)	C26—C27—O29—C30	179.3 (3)
C15—C16—C17—C22	−16.3 (7)	C27—O29—C30—C31	173.4 (4)
C16—C17—C18—C19	179.8 (4)	O28—C27—O29—C30	−1.1 (6)

### (II) trans-Dichlorido(4-chloroaniline-κN){4-ethoxycarbonylmethoxy-3-methoxy-1-[(2,3-η)-prop-2-en-1-yl]benzene}platinum(II) Hydrogen-bond geometry (Å, º)

**Table:**

*D*—H···*A*	*D*—H	H···*A*	*D*···*A*	*D*—H···*A*
N2—H2*AA*···O23^i^	0.91	2.49	3.025 (5)	118
N2—H2*AA*···O25^i^	0.91	2.47	3.377 (5)	174
N2—H2*AB*···O28^ii^	0.91	2.22	3.085 (5)	158
C26—H26*A*···Cl9	0.99	2.73	3.581 (4)	144

Symmetry codes: (i) *x*, *y*+1, *z*; (ii) −*x*+1, −*y*, −*z*+1.
